# A Survey on Human Cancer Categorization Based on Deep Learning

**DOI:** 10.3389/frai.2022.884749

**Published:** 2022-06-27

**Authors:** Ahmad Ibrahim, Hoda K. Mohamed, Ali Maher, Baochang Zhang

**Affiliations:** ^1^Department of Computer Science, October 6 University, Cairo, Egypt; ^2^Department of Computer Engineering, Ain Shams University, Cairo, Egypt; ^3^School of Automation Science and Electrical Engineering, Beihang University, Beijing, China

**Keywords:** human cancer, medical imaging, deep learning, convolutional neural network, cancer types

## Abstract

In recent years, we have witnessed the fast growth of deep learning, which involves deep neural networks, and the development of the computing capability of computer devices following the advance of graphics processing units (GPUs). Deep learning can prototypically and successfully categorize histopathological images, which involves imaging classification. Various research teams apply deep learning to medical diagnoses, especially cancer diseases. Convolutional neural networks (CNNs) detect the conventional visual features of disease diagnoses, e.g., lung, skin, brain, prostate, and breast cancer. A CNN has a procedure for perfectly investigating medicinal science images. This study assesses the main deep learning concepts relevant to medicinal image investigation and surveys several charities in the field. In addition, it covers the main categories of imaging procedures in medication. The survey comprises the usage of deep learning for object detection, classification, and human cancer categorization. In addition, the most popular cancer types have also been introduced. This article discusses the Vision-Based Deep Learning System among the dissimilar sorts of data mining techniques and networks. It then introduces the most extensively used DL network category, which is convolutional neural networks (CNNs) and investigates how CNN architectures have evolved. Starting with Alex Net and progressing with the Google and VGG networks, finally, a discussion of the revealed challenges and trends for upcoming research is held.

## Introduction

Cancer is considered the foremost cause of death globally (Mattiuzzi and Lippi, 2019). Both doctors and researchers face the challenges of fighting cancer. The World Health Organization (WHO) estimates that by 2040, the number of cancer cases may increase to 27.5 million, resulting in about 16.3 million expected deaths.

Early cancer detection is the highest priority for many people to save their lives (Maine et al., 2011). For the types of cancer diagnosis, visual inspection and manual procedures are typically used. This interpretation of medical images is error-prone and time-consuming.

As such, starting first in the 1980s, computer-aided diagnosis (CAD) systems were used to support doctors to enhance medical image interpretation efficiency (Sellars, 2022).

The leading phase of machine learning implementation is feature extraction. Different feature extraction methods for different cancer types have been investigated. However, there are many weaknesses in these feature extraction-based methods. To overcome these limitations and improve performance, learning was proposed in Georgiou et al. (2020). Graphics processing units (GPUs) were applied in parallel deep learning intended for image recognition and feature extraction (Gavali and Banu, 2019; Fan, 2021). For example, cancer detection was achieved through convolutional neural networks with a promising performance as in Yoo et al. (2019) and Hassan et al. (2020).

Deep learning (DL) is a novel subfield of machine learning that was stimulated by the humanoid mind's construction (Strauß, 2018; Bhatt et al., 2021). By education from the bottomless, layered, and hierarchical reproductions of data, deep learning procedures can outperform old-styled machine learning reproductions. A few years ago, with the development of deeper learning procedures, several research teams succeeded in applying more complex classification models. DL is mostly used for image analysis of cancer in many applications, such as usual and unusual tumor classifications (Boyd, 2020).

This study aims to give a comprehensive overview of (almost) all fields in the application of DL methods for human cancer detection. Furthermore, it offers a dedicated discussion section covering ultramodern and open challenges, and an overview of research directions and technologies that have become important nowadays.

The contribution of this study is introducing the recent dramatic change in DL for human cancer diagnosis in medical imaging. Besides presenting a recent DL technique, research, medical picture technology, and well-known cancer datasets were used for multiple DL approaches and training in most important human cancer types. Furthermore, according to their structural design and learning approach, this research has proposed a mild classification for deep networks. The chief drive of this study is to cover the track for future researchers to figure out a roadmap for DL contributions in cancer detection.

The rest of the survey is laid out as follows: Section VISION-BASED DEEP LEARNING SYSTEM introduces the main DL algorithms used for medical image analysis, which are then referenced throughout the survey; the pathophysiology of most frequent cancer types is discussed in Section CANCER OVERVIEW; Section CATEGORIES OF IMAGING PROCEDURES IN MEDICATION goes into the discovery of medication imaging procedure categories; the article concludes with a summary, a critical dialogue, and a forecast for future research.

## Vision-Based Deep Learning System

The vision-based deep learning system (VBDLS) is one of the harvests of human-inspired artificial intelligence (Rasouli, 2020). It presents an interest in two main research areas: computer vision and machine learning, where advanced machine learning techniques solve computer vision tasks more efficiently.

This section is organized to reflect an understanding of deep learning applications in the development of medical imaging systems. It consists of three parts: first, spotting the light on different methodologies of deep learning; second, categorizing the VBDLS according to its architecture and objective function; finally, presenting common deep network architectures and figuring out their potential and limitations.

### Learning Methodologies for VBDLS

In the VBDLS, learning is conducted utilizing a dataset that comprises a massive number of images. The traits of dataset besides the scope or space of the application judge the learning process in one of three main methods as follows: supervised, semi-supervised, and unsupervised.

There are also other approaches to learning, namely, reinforcement learning (RL) and deep RL (DRL), which are frequently discussed under semi-supervised or unsupervised methods.

#### Supervised

As a rule of thumb, a labeled dataset is used in supervised learning (Cunningham et al., 2008; Esfahlani et al., 2022), where the case problem to solve has a set of input *x*_*t*_ and corresponding known output *y*_*t*_.

In such an approach, the deep network predicts $\overset{ˇ}{y_{t}}=f\;\left(x_{t}\right)$, for input *x*_*t*_, the network receives a loss value of $L\left(y_{t},\;\overset{ˇ}{y_{t}}\right).$ Consequently, the trained network will iteratively adjust its parameters (weights) by itself for a better prediction of the actual output *y*_*t*_. After completing appropriate training, the network will be able to get correct responses to right inquiries, i.e., is the detected cancer from the medical image under examination benign or malignant?

#### Semi-supervised

Learning that takes place based on partly labeled datasets is called semi-supervised learning (Van Engelen and Horqos, 2020; Umamaheswari and Babu, 2021). DRL and generative adversarial networks (GANs) are the most well-known networks that exploit semi-supervised learning scenarios. There are basic distinctions between semi-supervised and supervised learning. First, users cannot have full access to the feature they are attempting to improve. so, they need to question it. Second, communicating with a state-based environment is through interaction: input *x*_*t*_ depends on earlier acts. In DRL, there is not a straightforward loss feature, making it harder to learn compared to traditional approaches under supervision.

#### Unsupervised

Unsupervised learning systems are those that can predict without the presence of data labels (Wahid et al., 2022). In this scenario, to discover unknown relationships, the network learns the internal representation or significant characteristics in the input data or structure.

The deep networks utilize unsupervised learning to accomplish data clustering, reduction of dimensionality, and augmentation via generative networks. Furthermore, autoencoders (AEs) and restricted Boltzmann machines (RBMs) are two members of the family of DL that are good at clustering and minimizing non-linear dimensionality.

The volume of training data available in the case of medical images is not that high. In addition, many labeled images are often difficult to obtain, as annotation itself is a costly task that is also scarce in databases for certain diseases (e.g., lesions).

### VBDLS Structural Categorization

A deep neural network can be categorized according to its structure “architecture” and learning method. This section will address well-known deep learning models; deep belief networks (DBNs), stacked autoencoders (SAEs), and convolutional neural networks (CNNs).

#### Deep Belief Networks (DBN)

**Figure 1:**
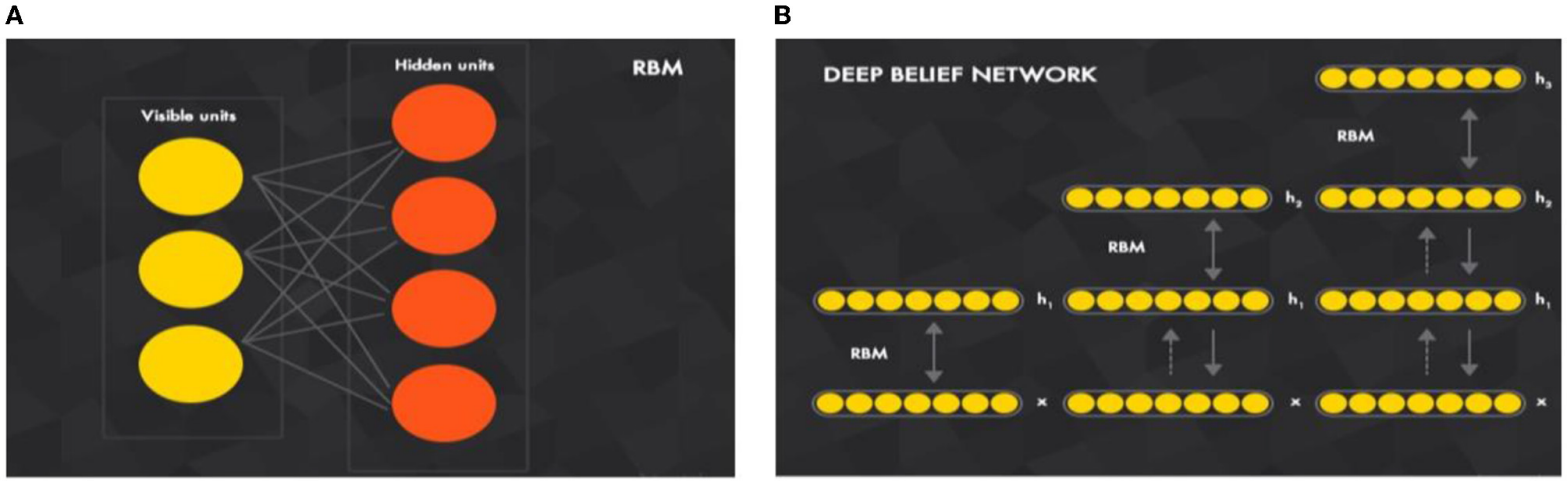
Key component of building a **(B)** deep belief network is a **(A)** restricted Boltzmann machine **(A)** (Ghosh et al., 2021).

A DBN is a generative model that comprises a set of restricted Boltzmann machine (RBM) layers as shown in Figure 1A. It is a two-layer (visible and hidden), bipartite, and undirected model (values can be propagated from visible-to-hidden and hidden-to-visible directions) that is fully connected (each neuron from a visible layer is connected to each neuron in the next hidden layer). If there is any connection in two neurons in the same layer, then a Boltzmann machine will be applied (rather than restricted Boltzmann).

The energy function of an RBM is used to infer its probabilistic meaning where the energy function can be used between the visible and hidden layer units to infer the conditional probabilities of inputs and outputs. In the forward and backward propagations, the probabilities **p**(**output**|**weighted**_**input**) and **p**(**input**|**weighted**_**output**) will be estimated. As such, it is called the generative model, because it is trained to reconstruct the input values, i.e., estimates the probability density function (pdf) of the input.

The RBM input vector **x** propagates through a weighted connection **W** to the output vector **h**, DBN is a set of RBMs connected in a sequence in which the training of DBN is conducted “individually” for each set. The undirected propagation of the exercise set begins with the first RBM. Then, for the second RBM, the first RBM will propagate the input through its trained weight to start undirected training and so on. As illustrated in Figure 1B, DBN is a joined set of RBMs. This idea of training individual RBMs in a sequence is to achieve different non-linear representations of input data at each DBN layer.

The energy function in vector form between visible neuron *v* and hidden neuron is given:


(1) $$\begin{array}{r} E\left(v,h\right)=-b^{\prime }v-c^{\prime }h-h^{\prime }W \end{array}$$

In this equation **h**′ and **v**′ are activations of hidden neurons and input data vectors, respectively. **W** is the weight matrix represented by connections between neurons, and *b* and *c* are biased vectors for visible units and hidden units, respectively.

The probability distribution function is given as follows:


(2) $$\begin{array}{r} p\left(v,h\right)=\left(e^{-E\left(v,h\right)}\right)/Z \end{array}$$

*Z* is a normalization constant defined as the sum of *e*^−*E*(*v, h*)^ over all possible configurations.

#### Stacked Autoencoder (SAE)

Autoencoders (AEs) are stacked in sequence to build SAEs for encoding (mapping) input data to other useful representations and then to try to decode (reconstruct) this representation again to the original input data. As a result, the reconstruction error (loss) between the original and recreated data is minimized. In other words, how encoded representation is efficient by decoding it and measuring the loss of data.

**Figure 2:**
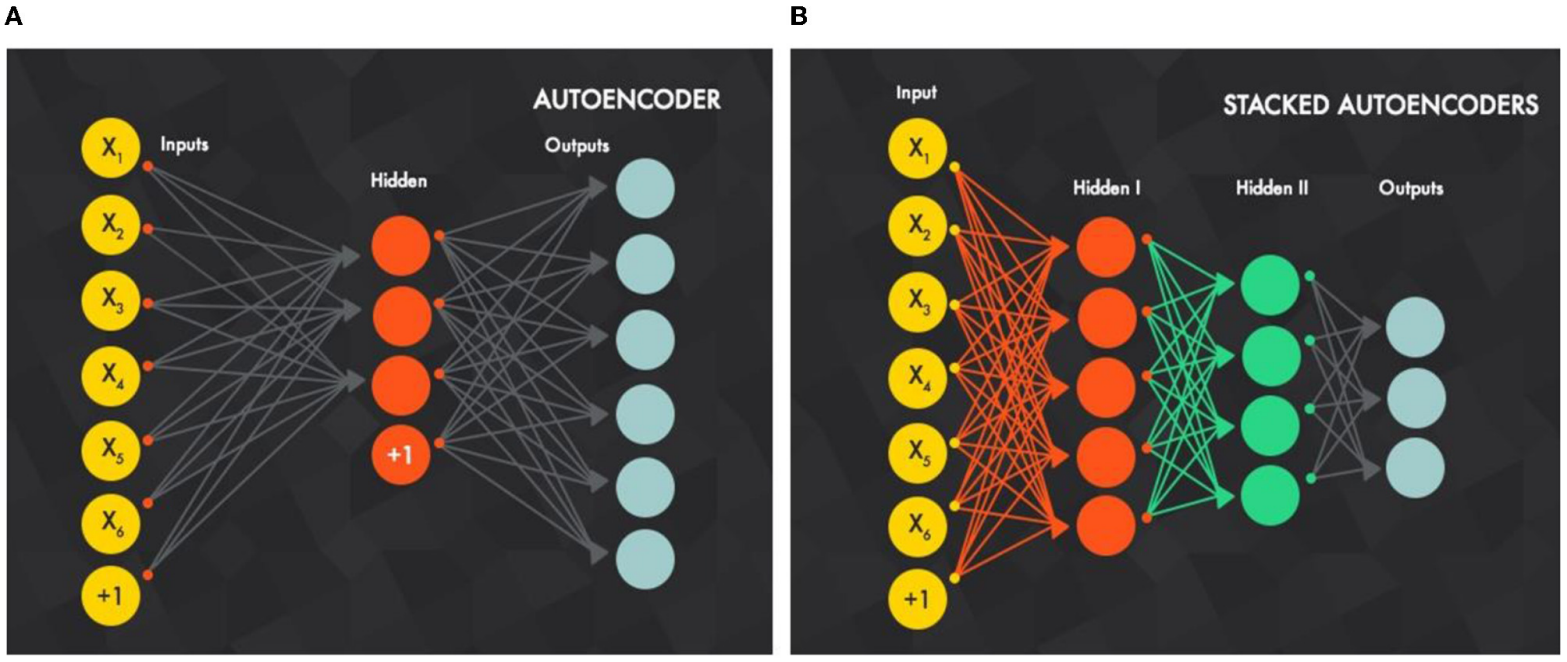
**(B)** Stacked autoencoders are built by **(A)** stacked autoencoders in sequence, best viewed in color (Xiang et al., 2016).

Figure 2 illustrates how an SAE is built by combining AEs. Like a DBN, the training is conducted layer-wise, but here it is directed from the input layer to the next layer. For the first decoding step (first AE) using the backpropagation method, the training is conducted individually with all available training data. For the second AE, the output layer of the first AE, which is the input layer of the second AE, is removed. Then, the input is clamped to the first AE input layer and propagates to the second AE output layer by all available training instances with backpropagation, and so on. For any upcoming AE, its input layer, which is the output layer from the previous AE, will be removed and will take the input from the previous AE input layer.

Intuitively, the number of hidden units is decreased as the decoding process goes more in-depth, because the SAE is forced to capture the most robust features from input data.

#### Convolutional Neural Network

Convolutional neural network (CNNs) have been used for more than two decades. From their name, the elementary image-processing task (spatial convolution) is conducted in the artificial neural network framework. At that proposed time, a CNN was not achieving noticeable efficiency in computer vision applications. The main reason it has now become the most successful one is the ability to go deeper with that network that reflects its great ability of high-level feature extraction. The hierarchical architecture of a deep network is utilized to extract distinct levels of features, from primary ones (low-level) to semantic features (high-level) like multi-level learning and mapping. In machine vision applications, low-level features, such as colors and edges, are extracted through previous layers and then summarized (pooled) and augmented with other low-level features as going deeper with network layers.

**Figure 3:**
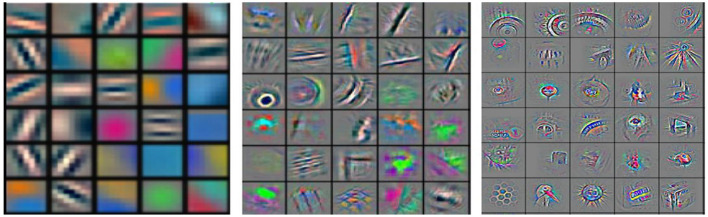
Different feature maps of a convolutional neural network [CNN, simpler from the left and more semantic (comprehensive) going (deeper) to the right] (Sarvamangala and Kulkarni, 2021).

Thus, high-level (abstracted) features can be obtained through the following layers. Abstracted features have a tremendous discriminative aptitude that will aid a simple classifier to achieve better performance in classification and regression tasks. Moreover, the different feature extraction levels (depicted in Figure 3) are conducted in an automated hierarchical structure through the convolutional neural network layers.

As shown in Figure 3 (on the left), shallower features are more straightforward, and (on the right) deeper ones are comprehensive and do not need professional manual design contrast with traditional “handcrafted” features. However, they require vast and more representative data compared to other methods and more computing capability of computer hardware for training them well and fast.

As mentioned above, the CNN's key element is the convolutional layer; each convolutional layer typically involves three stages:

- Convolutional stage: using a set of kernels (filters) to convolve them with an input image or the output from the previous convolutional layer, the output of the convolving process is called the feature map. Any filter captures the features by giving a high response with a similar sub-window to it and with the response going lower as it differs from the convolved sub-window. Each filter will produce one feature map, and it differs from other filters in the same layer. Thus, the number of output feature maps for each convolutional layer is equal to the number of kernels. The shared weight concept coming from each filter will be convolved with the input to produce a feature map in the sliding window method, so that a filter parameter (convolution layer weight) will be shared (convolved) with all input units.
- Detector stage (non-linearity): the activation function gives the non-linearity representation for the input and usually a rectified linear unit (ReLU) is used in a CNN.
- Pooling stage: this is considered the summarization stage (sub-sampling), which summarizes the output response of the kernels after mapping in the detector stage to a down-sampled feature map.

**Figure 4:**
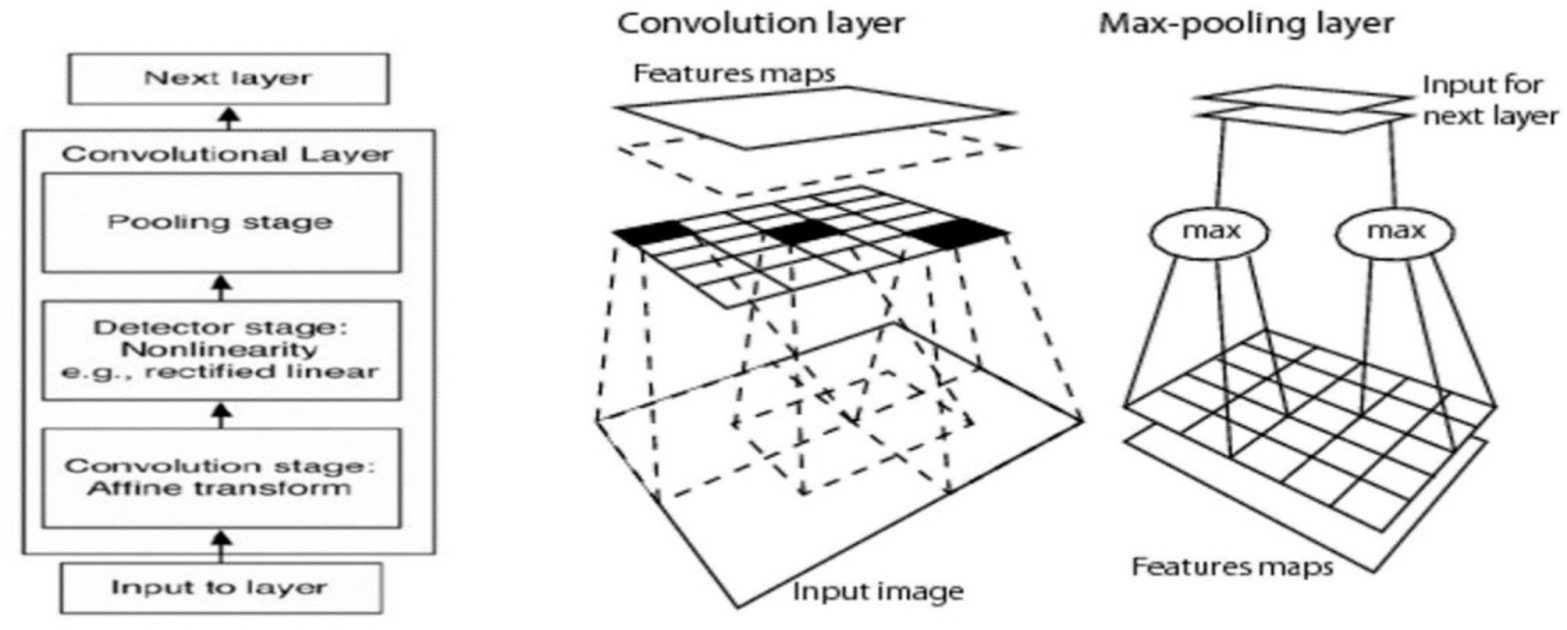
Key element of CNN architecture is the convolutional layer that involves convolution, activation, and pooling (Sarvamangala and Kulkarni, 2021).

Figure 4 illustrates how a CNN was built by combining convolutional layers. A CNN deals with spatially correlated data (e.g., images) where kernels work in the sliding window technique to capture local features (have spatial connectivity).

It is worth mentioning that the last layer is often a fully connected one to achieve a fixed vector dimension that represents the feature vector of the input data. Then, it can be used to train a simple classifier like a support vector machine.

Table 1 mentions the most flourishing and extensively used implementing codes for deep network key elements and architectures.

**Table 1:** Some of the existing codes online.

**Method Name**	**Online Code Link**
Restricted Boltzmann Machine	https://github.com/echen/restricted-boltzmann-machines
Convolutional	https://gist.github.com/JiaxiangZheng/a60cc8fe1bf6e20c1a41abc98131d518
Neural networks	https://github.com/siddharth-agrawal/Convolutional-Neural-Network
Multi-scale CNN	https://github.com/alexhagiopol/multiscale-CNN-classifier
Learning CNN	https://github.com/yanyongluan/MINNs

#### Common CNN Architectures

One of the first deep networks, the Alex Net (Khan et al., 2020) architecture, was used to improve the accuracy of ImageNet classification to a great extent compared to conventional methodologies. Five convolutional layers were included in the architecture, followed by three fully connected layers. By substituting activation parts, such as tanh or sigmoid functions, the ReLU activation function for the non-linear portion was presented. A ReLU has fast convergence compared to activation functions, which suffer from the vanishing gradient problem.

The visual geometry group (VGG) (Muneeb, 2018) at Oxford University suggested the VGG 16 architecture. By altering the size of kernels and introducing several filters, the VGG improved the Alex Net architecture. Large kernel-sized filters are replaced with multiple 3-3 kernel-sized filters (i.e., 11-11 in Conv1 and 5-5 in Conv2) that are placed one after another. Compared to a larger kernel size, several smaller kernel filters enhance the receptive field, as multiple non-linear layers increase the depth of a network. The increased depth allows for more complex features to be learned at a lower cost.

Although the VGG has achieved an exceptionally good accuracy in classification tasks for the ImageNet dataset both in terms of storage memory and time, it is computationally costly and requires enormous computational power. Thus, because of the large width of convolutional layers, it is inefficient.

Google Net proposed the idea that because of correlations between them, the vast majority of links between dense architecture and deep network activations are redundant. This makes it computationally costly for a network. Google Net, therefore, implied that a network with sparse connections among activations was the most efficient.

The initiation module was introduced by Google Net, which effectively calculates the sparse activation in a CNN with normal dense construction. The network also uses three different convolution sizes to increase the receptive field and retrieve features from extremely tiny levels (i.e., 5-5, 3-3, and 1-1). One of the significant highlights of the inception module is that it also has a so-called bottleneck layer (1-1 Conv.) that helps to massively reduce the computational requirement.

The global average pooling at the latter convolutionary layer is another change that Google Net introduced, consequently averaging the channel values across the 2D feature map. This results in a reduction in the entire sum of parameters. The accuracy of the network is saturated and consequently reduces quickly by increasing network depth. This reduction is not triggered by a problem of overfitting, but the training error also increases with the addition of more layers, leading to a problem of degradation.

The reduction problem was resolved by the introduction of the residual network (ResNet).

To study the training parameters effectively in a deeper network, the residual module was introduced. In a block-wise manner, a skip connection in convolutional layers to construct a residual module was introduced. ResNet performs better than VGG and Google Net (Khan et al., 2020).

## Cancer Overview

### Lung

**Figure 5:**
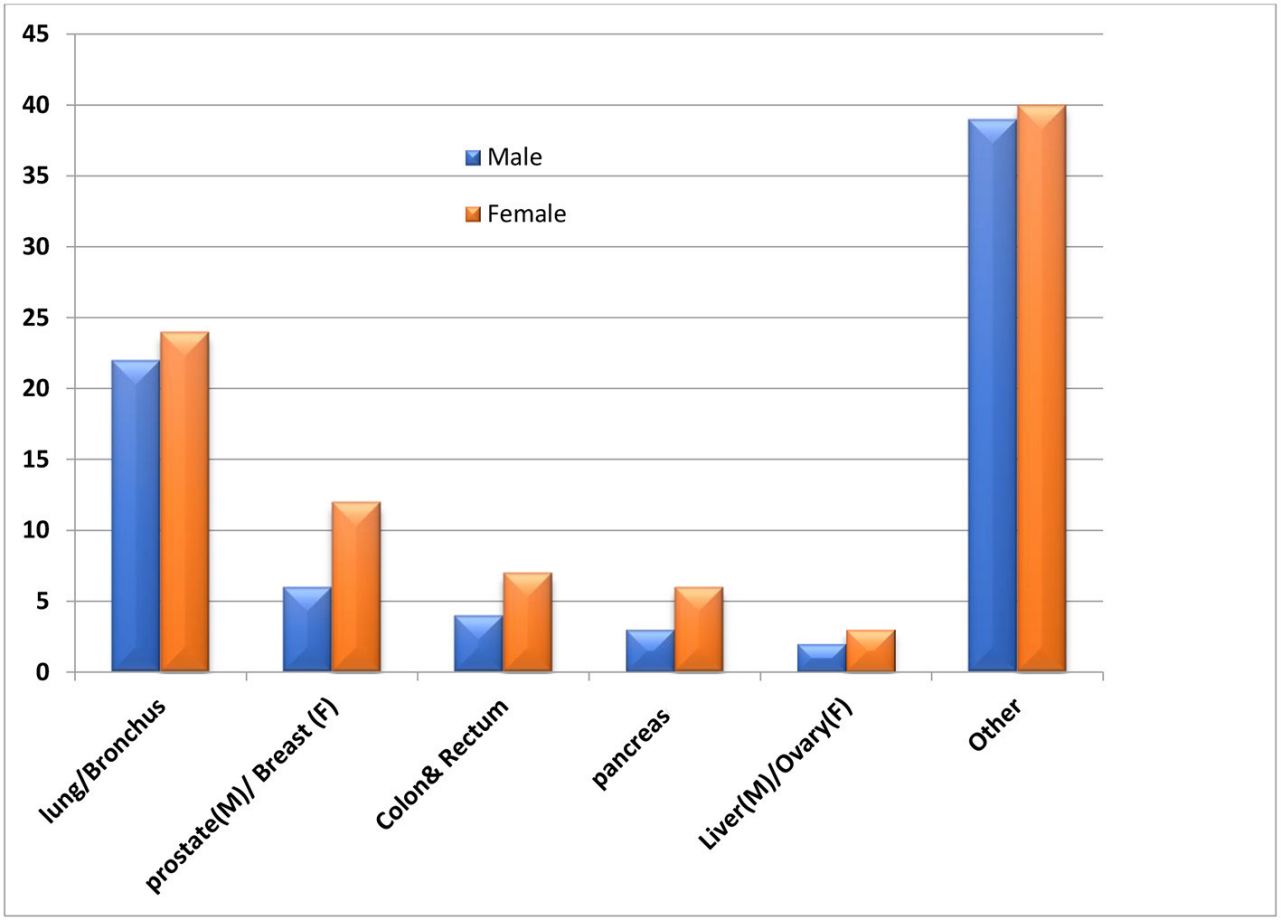
Average percentage of annual deaths due to various cancer types, best viewed in color (Munir et al., 2019).

Lung cancer causes a considerable number of deaths annually, as illustrated in Figure 5. Patients' survival time was effectively expected from their lung cancer pathological images using the deep network's detection efficiency. A pre-trained CNN (Singh et al., 2020), on big-dimension data, was intended for the classification of lung cancer by extracting features from CT images. A CNN and a deep belief network (DBN) were used in the classification lung of the raw image with an end-to-end model. A set of 2D CT images has been spawned from a patient's 3D CT image to ingest a multi-view CNN for an end-to-end training process. The extracted features for each 2D patch are concatenated to use their discriminative power and ease the classification task (Han et al., 2021). Consequently, a study was introduced (Yang et al., 2016) to deal with 3D images directly as an alternative to representing them in a 2D model based on a 3D CNN architecture.

A multivariant CNN (Mv-CNN) was presented in Cao et al. (2020). This prototype was considered to overwhelm the issue of variable nodule size. In the CNN model, the max-pooling layer is replaced by the multi-crop pooling layer to create multi-dimension features. In the non-linear transform, a randomized leaky rectified linear unit (RReLU) was used. Node-centric visual features can benefit from a multi-crop pooling method, where the standard max pool is useful for feature subclass selection and feature map size reduction. As a result, the pooling procedure reduces the characteristics by one level. A repeat pooling strategy is employed in multi-crop pooling, allowing the system to achieve multi-dimension features. **Figure 7A** depicts the accuracy of the most common lung cancer nodule classification architectures: DCNN, ResNet, optimal deep network, and VGG-16. An ensemble method for 3D-DCNN could more successfully capture the features of spherical-shaped nodules. DCNNs were trained using 62,492 samples from the Lung Image Database Consortium, including 40,772 nodules and 21,720 non-nodules. While using the ResNet, the accuracy of malignancy classification was prejudiced by curriculum learning, transfer learning, and varying network depths.

### Breast

In the last years, in breast cancer, many studies have been conducted for recognition and diagnosis. The proposed technique (Elazab et al., 2020) employs deep learning for recognition of mitosis in histopathological images of the patient's breast. Features had been extracted from a trained CNN and then fed to a support vector machine (SVM) to classify mitosis of the infected breast. The well-known Alex Net is picked, as it has the CNN architecture that achieves fair results for pathological image classification task.

For detection of mitosis from breast histology slides, a deep cascade network (DCN) was proposed (Zhou et al., 2020). Mitosis candidates were segmented from histology slides using a fully convolutional network (FCN) model. Then, a pre-trained Caffe Net model (Munir et al., 2019) on the ImageNet dataset was finely tuned for mitosis classification. Then, a network with various configurations for three networks with fully connected layers was trained. Then, the results were collected as a shape of many results of probabilities. Then, the average for these outputs was taken, and the last results were generated.

A multi-scale CNN architecture was deployed (Rahman et al., 2020) to analyze breast histopathological images. The network structural design is based on using an aggression layer (AL) after every soft max layer (SL) to collect the estimated outcomes from various members with annotation outcomes. A stacked sparse autoencoder (SSAE) is deployed to classify breast nuclei in histological images (Xiang et al., 2016). The proposal applies the greedy strategy to accomplish SSAE optimization by training each hidden layer separately.

For recognizing the mass of digital mammograms as in a proposal (Zhou et al., 2019), a trained CNN alongside the SVM was introduced. The production of the last fully connected layer stands for the input of high-level feature representation, and mammogram areas were applied for the training of the CNN model. Then, the classification was conducted using an SVM that has been trained by high-level feature representation. A transfer learning strategy was used to train the CNN model. The mass of obtainable mammograms could be identified by applying a CNN.

Overfitting occurs when a reduced sample is used in training, which leads to a low bias/high variance model that cannot generalize well to the test data. In this context, a proposal (Williams and Rodriguez, 2020) applies procedures to enhance training data and defeat over-fitting by deploying statistical self-similarity and non-negative matrix factorization (NMF). A promising model (Ghosh et al., 2021) that initially discovers the existence of a mass as a preliminary step for its detection from mammography was proposed. This model considers sparsity regularization to realize the features of mammograms in several ranges using a stacked convolutional sparse autoencoder (SCAE).

Different potential functions were combined through a structured SVM for mass segmentation in mammograms and included a Gaussian mixture model, before location, and a beep belief network (DBN). From the same perspective, the proposed model (Dhungel et al., 2015) uses the cascade of random forest classifiers and DL for mammogram mass detection and recognition.

To learn the bilateral features from the Digital Breast Tom (DBT) synthesis, there are three-dimensional multi-views introduced in the model (Kim et al., 2015). A volume of interest (VOI) was achieved from the source volume, which was managed in the registered target as an individual input rather than a VOI. Two individual CNNs were used to extract high-level characteristics from these two individual VOIs.

**Figure 6:**
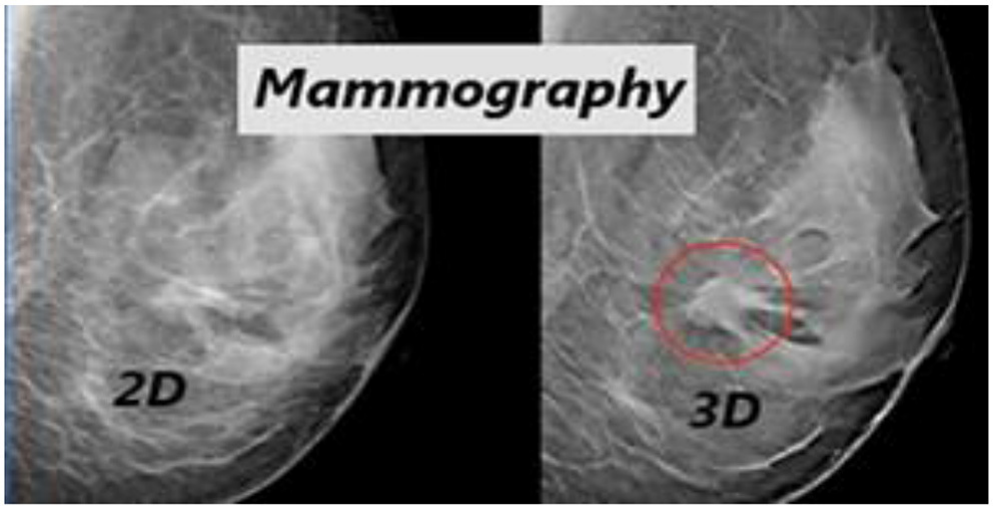
Qualitative difference between the 2D and 3D (tomosynthesis) mammography for breast cancer diagnosis (Zhou et al., 2019).

**Figure 7:**
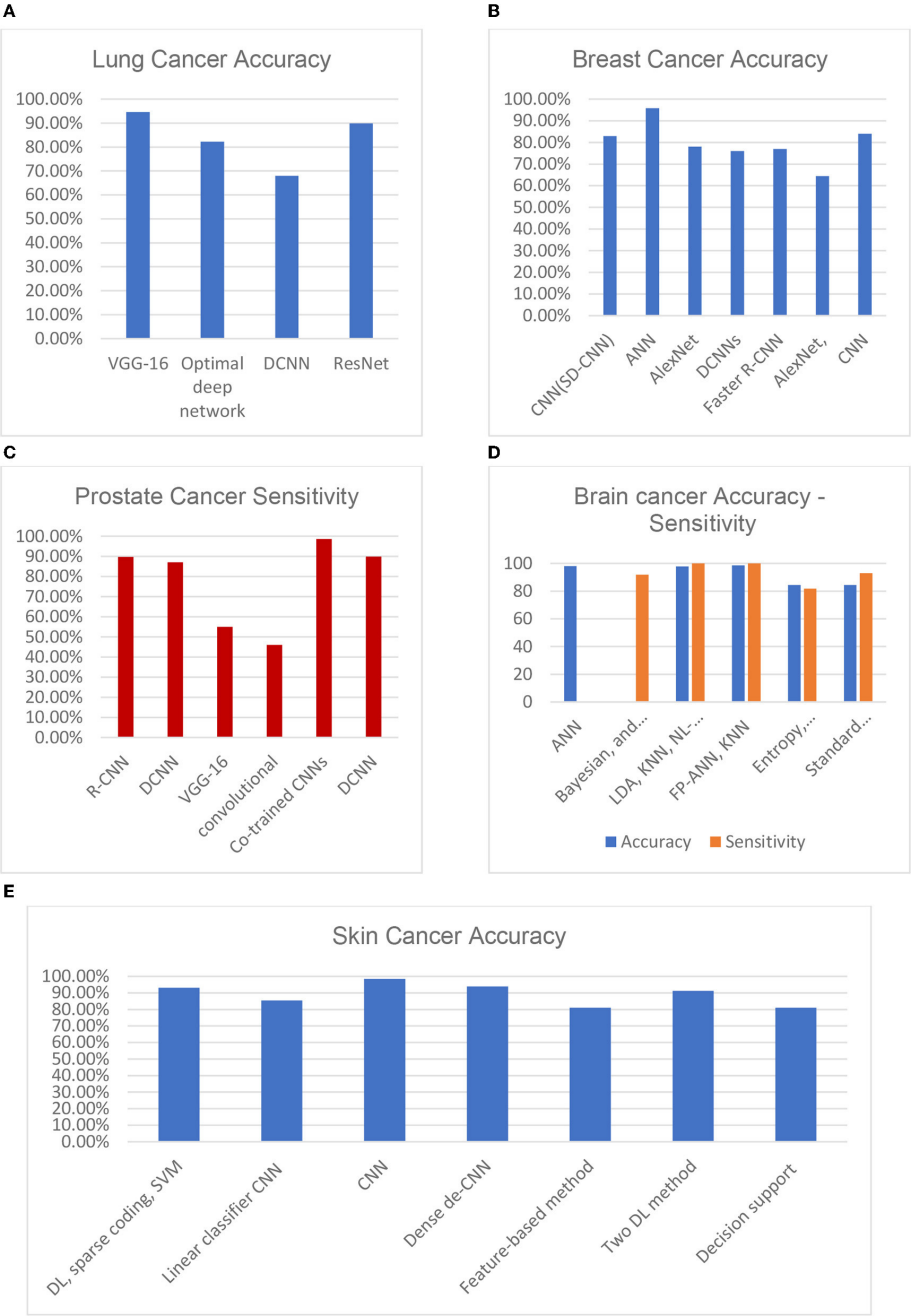
Various DL approaches' recent performances in terms of their diagnosing accuracy for **(A)** lung cancer, **(B)** breast cancer, **(C)** prostate cancer, **(D)** brain cancer, and **(E)** skin cancer (Munir et al., 2019).

Figure 6 illustrates the qualitative difference between the 2D and 3D (tomosynthesis) mammography (Iranmakani et al., 2020) for breast cancer diagnosis. Figure 7B depicts the accuracy of many studies that investigated the use of CAD systems for breast cancer detection, employing a variety of medical imaging modalities and CNN-based methods. Using CNNs for a model trained on 540 images, an accuracy of 95.8 percent was obtained, resulting in a segmentation-free result. Results with an accuracy of 78.1 percent when using a 3D-CNN demonstrated that 3D-CNN methods could be a promising technology without manual feature extraction. The DCNN architecture was compared to CNN architectures. CNN and DCNN had AUCs of 0.89 and 0.93, respectively.

### Brain

Brain cancer is the uncontrolled growth of cells in any part of the brain. Detection of which area of the brain holds cancer is quite difficult. Therefore, segmentation of the brain from the healthful part is the major challenge for brain cancer.

Two algorithms built on 2D CNN and 3D CNN were introduced (Gao et al., 2017) starting with 2D sliced images and 3D images. The output from these two models was fused to the result. The 2D/3D scale-invariant features (SIFTs) and Kaze are outperformed by this hybrid deep model.

The adjacent patches for the brain picture were connected using a dense training technique in the CNN (Sarvamangala and Kulkarni, 2021). The false positives were eliminated using a 3D fully linked random field, and the images were then segmented in 3D using the CNN. The proposed approach combines multi-modality data from T1, T1C, T2, and fluid-attenuated inversion recovery (FLAIR) images and usess this information to train the suggested CNN. The system suggested 3D voxel categorization based on a well-trained CNN. Various-sized 2D patches were created by splitting the 3D dataset into 2D slices. For the learning procedure, the sliced 2D patches were fed to numerous CNNs. Figure 7D depicts a variety of ML-based brain cancer classification system barograph of sensitivities. The obtained accuracy for ANN-based classifier on features extracted from 100 T2 weighted MRI images using Discrete Wavelet Transform (DWT) was 98 percent. While SVM achieved a sensitivity of 91.84 percent when applied to 14 DWI, (B), fluid-attenuated inversion recovery (FLAIR), T1, and GAD images. Using an NL-SVM on SVM-RFE features, a 97.8 percent accuracy was obtained from 102 T1, 2 FLAIR, and relative cerebral blood volume (RCBV) images. FP-ANN and KNN were applied to features extracted using discrete wavelet transform from 70 MR images and achieved a maximum accuracy of 98 percent. Gray level co-occurrence matrix (GLCM) features extracted from 42 diffusion-weighted images, apparent diffusion coefficient images, using entropy histogram techniques yielded an accuracy of 84.4 percent. An accuracy of 95 patients' standard deviations from 95 T1W, T2, and FLAIR images was accomplished in the range of 84.4 percent. A summary of well-known datasets for cancer types is given in Table 2.

**Table 2:** Summary of well-known cancer datasets that are used for deep learning (DL) approach training.

**Cancer type**	**Dataset name**	**Cases**
Breast Cancer Classification	BreakHis (Zhou et al., 2020)	9,109 microscopic images were collected from 82 patients
Lesion recognition	DDSM (Martin et al., 2018)	2,620 scanned film mammography
Lung nodule	JSRT (Yang et al., 2016)	247 images each: 154 cases with lung nodules and 93 cases without lung nodules
Lung nodule-Dishonesty classification	LIDC (Cao et al., 2020)	1,018 instances
Melanoma detection	MED-NODE (Mohan et al., 2020)	170 images, 100 naevi, and 70 melanomas
Prostate Segmentation	PROMISE12 (Jia et al., 2018)	7,040 pictures
Brain cancer Segmentation	BraTS (Gao et al., 2017)	220 HGG and 54 LGG

### Skin

Several variables can raise a person's risk of acquiring melanoma. UVRs, sunburns, blisters, tanning, tanning salons, and sunlamps are all causes that occur before the age of thirty. Furthermore, there are risk factors that are not related to age, such as having two or more cases of melanoma in the family, having easily burned skin, and so on. Many methods, including the best-known ABCD rule, seven-point checklist technique, Menzies technique, and pattern analysis, have been applied.

A total of 399 images have been used to classify benign melanoma naevi in a proposed study (Khatib et al., 2020). Pre-processing and data increase were initially performed. High-level skin lesion characteristics have been extracted using a pre-trained CNN and Alex Net. K-Nearest Neighbor was used for lesion classification, achieving an accuracy of 93.62%. The binary classification model (Boman and Volminger, 2020) uses 129 and 450 images, the first classification distinguishes benign naevi from malignant melanoma: and the second classification distinguishes benign seborrheic keratosis from keratinocytes carcinomas. A total of 2,032 pictures of skin cases and the remaining images from dermatoscopic instruments were used for the retraining of the CNN. Transfer learning had been applied to the classification. The area under the curve (AUC) achieved was 0.96 for carcinomas and melanomas. For deeper extraction and classification of lesions, a pre-trained CNN with Alex Net and VGG 16 has been used. Through 19,398 images for training a Res Net model, the authors introduced a classifier model for classifying 12 distinct kinds of skin diseases. The AUC for squamous cell carcinoma was achieved through the Asan data set at 0.83 for intraepithelial melanomas and basal cell carcinomas, and 0.82 for intraepithelial carcinoma. Although pre-trained CNNs are present, efforts have been made to develop new CNN algorithms.

From the same perspective, a research study (Mohan et al., 2020) achieved an accuracy of 89.5% for skin cancer classification using 900 images and applying a backward-propagation technique in an eight-layer CNN model.

Three datasets were implemented for training the neural network model, with 888, 2,750, and 16,691 images. The authors in Tschandl et al. (2018) suggested a system built on content-based image retrieval (CBIR) in comparison with CNN applying SoftMax and using two performance measures and the AUC and (multi class-accuracy and mean average prediction, MAP). The third dataset results were superior, achieving an AUC value of 0.852 and an MAP value of 0.847.

For the classification of lesions with the CNN model together with ANN, the data set given by ISIC in the 2016 challenge was used. First, segmentation of images was performed using the intensity threshold, and then the CNN extracted the properties. To conduct the classification, the ANN classifier applied these features.

An improved classification technique using a CNN was proposed with the method of data enhancement (Mikolajczyk and Grochowski, 2018). Furthermore, there were tries to overwhelm the data limitation problem and its consequence on the performance of the classifier. Six hundred test pictures and 6,162 for training were included in the dataset. The AUC value was 89.2%, ACC value was 89%, and AP value was73.9%. Analyzing the effect of image augmentation on three classifiers leads to better results compared to the typical techniques used in advance. DL methods have been applied to diagnose four skin diseases. A hierarchical structure was built to produce a summary of classification and diagnosis criteria. An accuracy of 87.25% was achieved with a probability error of 2.24%.

A study that uses a convolutional neural network for recognition of esophageal cancer, squamous cell carcinoma (SCC), and adenocarcinoma was introduced (Syed et al., 2020). A total of 8,428 images from 384 Japanese patients were used in the training pictures used in this study. The test data included 1,118 images for 47 patients suffering from esophageal cancer and fifty patients who do not have esophageal cancer. The precision achieved was 98%. Forty percent of every image was positive, while 95% was negative because of the presence of shadows, which was the cause of misdiagnosis. A study has been submitted to detect rose-shaded, flat leg lesions in elderly people (Martin et al., 2018). With the clinical diagnostic system, a precision of 49.1% was achieved. Figure 7E depicts a comparison score of the accuracies of skin cancer DL architectures. The result (95 percent accuracy) obtained on the PH2 database is better than the result (81 percent accuracy) obtained on the same database.

### Prostate

A combination of sparse patch matching and deep feature learning prostate segmentation was used to obtain a feature representation in MR images from the SSAE approach. The SAE classifier was used for the recognition of prostate cancer. By checking the way to fine-tune the SSAE model, the collected features were improved.

The reconnaissance map was refined using the neighbor pixel relationship energy minimization procedure. For prostate segmentation, the author (Tian et al., 2018) used a full CN. The authors segmented the prostate with images of 3D MR through volumetric CN. To enable the volume-to-voltage prediction, the FCN was extended with residual blocks. A patch-based CNN method was introduced in Jia et al. (2018) for using the region of focus and prostate cancer detection. By multi-atlas label fusion, the final segmentation result was achieved. Figure 7C compares the statistical sensitivity score of various CNN architectures on a prostate cancer dataset. The R-CNN framework for multi-task prediction with an epithelial network head and a grading network head achieved a 99.07 percent accuracy and an average AUC of 0.998. On ImageNet, AUCs of 0.81 and 0.83 were obtained using V3 and VGG-16, respectively. Two prostate cancer diagnostic tasks are handled with a multimodal CNN. The proposed network was used in the first phase to classify cancerous and non-cancerous tissues, and in the second phase to differentiate clinically significant prostate cancer and indolent prostate cancer. The results show that prostate tissue classification has a sensitivity of 89.85 percent and a specificity of 95.83 percent, and that the prostate cancer characterization has a sensitivity of 100 percent and a specificity of 76.92 percent.

## Categories of Imaging Procedures in Medication

In the analysis of cancer images, imaging is the first step for abnormality detection. There are numerous methods for detecting anomalies, decomposing, classifying, denoising, and diagnosing diseases from medical images. The most often utilized techniques are CT scans, radiography/funds (e.g., X-ray and CFI), microscopy, ultrasound, magnetic resonance [(f/s) MRI], and positron emission tomography (PET). DL and RL architectures have shown to be more successful than others. Image denoising is a key factor for the success of several medical image analysis approaches, so in image analysis, a CNN has been the communal architecture of DL and easing structure. A CNN was used in many image classifications like different neuroimages and mammograms (MMM). Because of colonic polyps and lymph nodes (LNs) in the spine, a CNN was used to detect clerotic metastases and anatomical structures by CT scan. Various medical images aid in the recognition of esophageal carcinoma and the forecasting of neoadjuvant chemical responses in patients with thoracoabdominal LN, interstitial lung disease (ILD), pulmonary nodules on (f)MRI, while diffusion tensor images to extract deep characteristics for brain tumor patients. PET images help to recognize esophagus carcinoma and forecast neoadjuvant chemical reactions. Furthermore, a DBN was positively useful to recognize care shortage hyperactivity disorder, while a DNN-based technique was suggested to positively recognize the fetal abdominal standard plane in ultrasound imaging.

### Medical Image Pre-processing

Conventional image processing tasks are conducted for acquired medical images before diagnoses, such as grayscale conversion and normalization. Grayscale conversion of medical images supplies only a gray tone, and the brightness for each pixel stands for its value in all channels. The dynamic range of all pixels is normalized by mapping it to another proper range for the next processing. Furthermore, various noises and artifacts may superimpose a medical image during acquisition and formation. The pre-processing task in this context is conducted to ease the diagnosing process by purifying the relevant features of the desired symptom from the irrelevant ones.

### Magnetic Resonance Imagining

**Figure 8:**
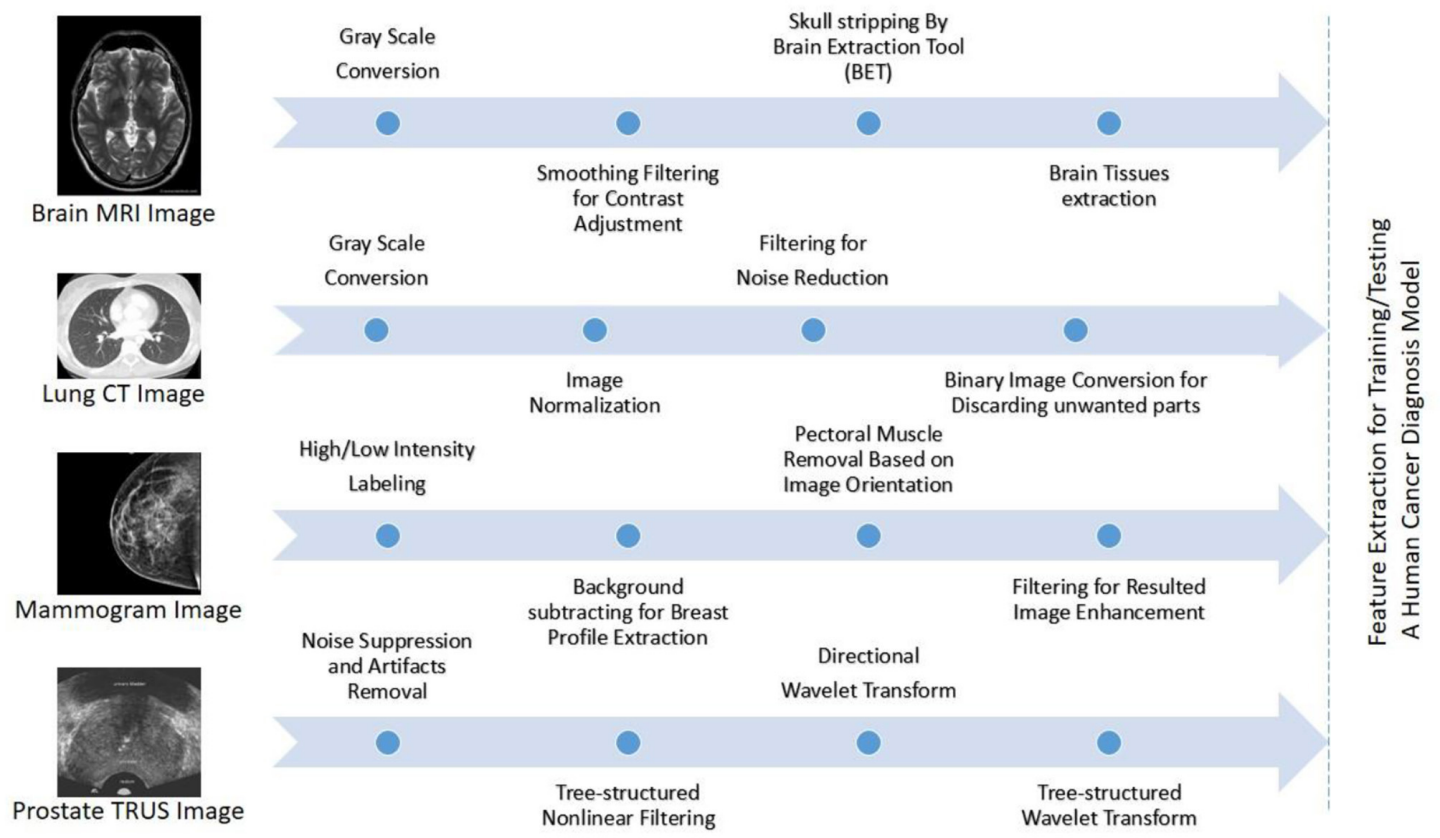
Typical flow graphs for the pre-processing tasks of well-known medical images: MRI, computed tomography (CT), mammogram, and transrectal ultrasound (TRUS) are illustrated from top to bottom, best viewed in color (Elazab et al., 2020).

MRI is applied in many areas like checking for breast cancer, clinical analysis, and the situation rise risk of patients. Many cases were proposed in earlier studies for MRI using CAD systems like breast abnormality classification, and MRI was dependent on breast division and breast abnormality detection. DCE-MRI (active contrast-improved MRI), a technically advanced form of MRI, has provided a cutting-edge volumetric resolution for cxwell lesion imaging and lesion temporal pattern improvement to cutting-edge information for well cancer organization. DCE-MRI has been shown in studies to be a useful tool for breast cancer diagnosis, prognosis, and linkage with genomes. In comparison to mammography and ultrasound, MRI has revealed a high level of sensitivity in detection of breast cancer. CE-MRI is a type of enhanced MRI that has been shown to have high sensitivity for cancer detection, even in dense breast tissues. Figure 8 shows a typical flow graph for the pre-processing tasks of well-known medical images: MRI, computed tomography (CT), mammogram, and transrectal ultrasound (TRUS).

### Ultrasound

In differentiation and breast lesion detection, ultrasound is applied because it is an imaging modality, but it is operator-reliant. Because of an operator who can utilize an ultrasound scanner to appropriately locate the case. As an alternative to DM, ultrasound imaging is used performed to detect and diagnose anomalies in breast cancer. Ultrasound was found to be quite accurate in detecting and distinguishing between benign and malignant crowds. This allowed imaging techniques in the United States to reduce the number of needless biopsies. Ultrasound was originally coupled with magnetic resonance imaging, digital mammography, and digital breast tomosynthesis imaging modalities because it was benign, precise, low-cost, and widely used. To interpret any specific lesion type, a thorough understanding of picture features is required, which makes ultrasound image interpretation difficult. Ultrasound is better suggested to be applied as an enhancement to DM due to its accessibility and affordability, likened to additional modalities, in addition, it is better tolerated by patients.

### Screen Film Mammograms

In the previous five decades, the standard imaging technique for detecting worrisome lesions in the early stages has been screen film mammography (SFM). SFM has a high sensitivity (100%) for detecting suspicious cases in breasts with fatty tissues (Selvi, 2015), and the reduction in lesion complicity could be due to the film itself, as it aids in image acquisition, display, and storage. Further augmentation is unlikely after the film is molded, and parts of the image may be exhibited with lower contrast. If image enhancements for photos with lower contrast are not possible, patients may request an additional mammogram, exposing them to extra radiation. Another drawback of the film is that different parts of the breast picture are classified according to the mammography film's characteristic response. Between active range (latitude) and contrast resolution, there is a compromise (gradient) (Henriksen et al., 2018).

### Digital Mammograms

It was the most valuable and standard method for breast imaging in the detection and diagnosis of breast problems. It does, however, have certain limitations, including low specificity. As a result, there may be a higher number of unnecessary biopsies, increasing expenditures and putting more burden on patients. Because of the lack of positioning of a deep tissue in cases when there is a lapping breast tissue, there is a high probability of missing some malignancies in the thereto-mammary area. The CAD system has found positive mammographic findings and is utilized in clinical routine to improve the radiologist's sensitivity. The CAD system, however, has three restrictions: high false-positive outcomes that suggest greater recall taxes, greater false-negative results, and great radiation exposure (Henriksen et al., 2018). A summary of DL publications for cancer diagnosis, detection, and prognosis is shown in Table 3.

**Table 3:** Summary of the most recent deep learning publications for cancer diagnosis, detection, and prognosis.

**Publication**	**Neural Network**	**Cancer type**	**No of patients/Images**	**Type of data**	**Reported Accuracy or AUC**	**Limitations**
Melekoodappattu et al. (2022)	CNN Ensemble system	Breast	644 individuals	^1^MIAS dataset.	98.00%	The model is limited to classify only two classes.
			2,620 images	^2^DDSM dataset	97.7%	
Li et al. (2022)	Multi class CNN	Lung	506 patients	Gansu Provincial Tumor Hospital dataset	83%	Did not compare the simulation results with other machine learning algorithms.
Elmarakeby et al. (2021)	DNN	Prostate	3,007 patients	Reactome pathway datasets	83%	The classification technique's performance was improved, however the confusion matrix and its performance measures were not estimated.
Ranpreet et al. (2021)	DCNN	Skin	10,070 samples	ISIC 2020	90.42%	The methodology requires real-time interfacing with medical images so that it can improve the medical field.
Lu et al. (2021)	CNN Marine Predators Algorithm	Lung	15,419 images	^3^RIDER dataset	93.4%	Did not measure other performances, e.g., precision, recall, Also execution time is not mentioned which can increase the value of the results.
Irmak (2021)	CNN	Brain	70,220 images	RIDER	99.33%	However, the method achieved a good result but results may vary for a different dataset.
			110,020 images	REMBRANDT		
			241,183 images	TCGA-LGG		

^1^*MIAS, Mammographic Image Analysis Society*.

^2^*DDSM, Digital Database for Screening Mammography*.

^3^*RIDER, Reference Image Database to Evaluate Therapy Response*.

^4^*ISIC, e International Skin Imaging Collaboration datastores*.

## Human Cancer Categorizations

Cancers are categorized according to their primary site of genesis and their histologic and tissue types (Bou Zerdan et al., 2022; Kim et al., 2022; Siegel et al., 2022).

### Categorization Based on Origin Position

Cancers could be categorized as per the main point of action, such as breast, lung, prostate, liver, kidney, and brain (Divate et al., 2022).

### Tissue Form Categorization

This categorization is depending on the International Classification of Diseases for Oncology, which divides malignancies into six categories depending on different tissues:

#### Carcinoma

The epithelial layer of cells that forms the covering of different exterior areas of the body or the interior linings of organs in the body gives rise to this type of cancer. Carcinomas, or malignancies of epithelial, account for 80–90% of all cases of cancer, because epithelial cells are found throughout the body, from the skin to the coating and covering of organs and inner passages like the intestinal system. Carcinomas most commonly affect secreting organs or glands, like the breasts, lungs, bladder, colon, and prostate. Adenocarcinoma and squamous cell carcinoma are two forms of carcinomas. Squamous cell carcinoma arises from a simple squamous, and carcinoma occurs in an organ or a gland. Mucus membrane adenocarcinomas are the most public sort of adenocarcinoma.

#### Sarcoma

Tumors of the connective and supporting tissues, such as muscles, bones, cartilage, and fat, are the source of these cancers. Osteosarcoma is a type of sarcoma that affects the bones. The young are the ones who are most affected. Sarcomas take on the appearance of the tissue, in which they develop into chondrosarcoma (cartilage cancer), leiomyosarcoma (smooth muscle cancer), rhabdomyosarcoma (skeletal muscle cancer), mesothelial sarcoma or mesothelioma (membranous lining of body cavities), fibrosarcoma (fibrous tissue), angiosarcoma (blood vessel cancer), or glioma (mixed connective tissue types).

#### Myeloma

Myeloma is produced in the bone marrow's plasma cells. In response to infections, plasma cells can produce a variety of antibodies. Myeloma is a form of cancer that affects the blood.

#### Leukemia

This is a type of tumor that falls under the category of blood cancer. This malignancy attacks the bone tissue, which is responsible for the generation of blood cells. When the bone tissue becomes malignant, it results in overabundance of immature white blood cells that are unable to perform their functions, leaving patients vulnerable to infection.

#### Lymphoma

Lymphocytic malignancies are tumors of the lymphatic system, as opposed to leukemia, which is a “liquid tumor” that affects the blood. Lymphocytic cancers are “hard malignancies.” They can affect lymph nodes in specific locations like the abdomen, brain, and intestines. Extranodal lymphomas are a type of lymphoma that occurs outside of lymph nodes.

Hodgkin's lymphoma and non-Hodgkin's lymphoma are the forms of lymphomas. Reed-Sternberg cells are found in hematological tissue samples; however, they are not seen in non-Hodgkin lymphoma tissue samples.

#### Mixed Types

There are two or many cancer components in these. Mixed mesodermal tumor, carcinosarcoma, adenosquamous carcinoma, and teratocarcinoma are only a few examples. Another form that incorporates embryonic tissues is blastoma.

### Categorization by Grade

Types of cancer can be categorized by their grade. Cancer is defined by abnormalities of tissues in relation to normal external tissues. The grade rises from one to four as the level of abnormality rises.

Well-differentiated cells are like normal specialized cells and are found in low-grade malignancies. Undifferentiated cells are severely aberrant in comparison to deeper structures. These are tumors with a high grade.

### Categorization by Stage

Individual cancers are also categorized based on their stage. Staging can be performed in a variety of ways. The most often used technique divides tumors into three categories: tumor size (T), regional dissemination or nodal involvement (N), and distant metastasis (D) (M). The TNM staging is what it is called.

T0 denotes no indication of tumor, T 1–4 denotes growing tumor size and involvement, and Tis denotes carcinoma (limited to surface cells). Similarly, N0 denotes no lymphadenopathy involvement, while N 1–4 denotes varying degrees of lymph node involvement. Nx denotes that node involvement cannot be determined. Metastasis has been further categorized into two kinds: M0 indicates that there is no indication of distant spread, and M1 indicates that there really is evidence of distant spread.

## Discussion

In the literature, gaps and limits in measurements are well-documented, highlighting a disconnect between DL researchers who create algorithms and physicians who make decisions. DL algorithms are mentioned as “black boxes.” Attempts to mitigate the algorithms' black box characteristics are required for a number of reasons. For starters, there are legal and ethical standards, as well as rules and regulations, that must be met before DL cancer detection systems may be used in clinical settings. The European Union's General Data Protection Regulation (GDPR), for example, requires enterprises that utilize patient data for classifications and recommendations to offer on-demand explanations. If organizations are unable to offer such explanations on demand, they might face severe penalties. DL models that can be explained are also linked to monetary incentives. Clinicians and patients must be able to trust the classifications provided by these systems, in addition to ethical and legal problems. An explanation is also essential for trust and openness. The goal of explanation techniques is to show the logic behind the model's classification, building trust between the system, clinicians, and patients. This can help to decrease the amount of inexact results that non-explainable systems can produce. Conclusively, explainable DL algorithms will provide clinicians with additional benefits like lesion detection and segmentation.

Besides, there are a quantity of limitations compared to outcomes that can help decision-makers. The first point to consider is taxonomy. Various taxonomies have been proposed to categorize explanation methodologies (Adadi and Berrada, 2018; Arrieta et al., 2020). Because the classification systems are task-dependent, there is no universally accepted taxonomy for clarification approaches. Clarification measures fall into the second type. The criteria for evaluating clarifying methods for cancer diagnosis may differ from those used for election prediction. As a result, it is necessary to evaluate the context of potential applications, in addition to basic testing of clarification methods. This is especially important for cancer-clarifying techniques because of the high-risk nature of predictions.

More study is needed to define what clinicians consider explainable, so that DL cancer detection systems may be compared to these findings. Another important possibility is to use findings to construct quantitative clarity metrics. The bulk of standards used to measure the superiority of clarifications is developed without the involvement of clinicians. It is vital to measure clarification strategies in a way that is led by end-user criteria to generate clarification approaches that are suitable for clinician integration. Clinically important features can also be extracted using clarification approaches. Although there have been research studies that use explanatory techniques to locate lesions, these models do not extract information, such as shape, volume, area, and other significant properties.

A future opportunity is to extract these features without computationally expensive segmentation. With this, clinicians do not need to extract these features manually. If clarification techniques are implemented in clinical settings, the system can automatically extract these characteristics for clinicians, thus aiding in the diagnosis process. The common approach to providing clarification for image classification is to produce a heatmap showing the most discriminative region. More studies are needed to be carried out to provide greater insights than using this direct approach. For example, it would provide more insights if clarification methods could explain why a classification was not made and quantify the uncertainty of the clarification. Being able to reason for and against is also important to provide greater insights and explainability.

Additional studies ought to be directed on where current methods fail and why. This would provide the community more insights on how to create more robust clarification methods. It would also provide greater confidence in the methods that are currently used.

## Conclusion

This study has discussed the recent significant shift in DL for human cancer diagnosis in medical images. The study has shed light on recent DL methodologies, research, medical image technologies, and well-known cancer datasets used for various DL approaches and training in most crucial human cancer types. Furthermore, this research has presented a gentle categorization of deep, according to their structural design and learning method. The main goal of this study is to pave the way for future researchers to create a roadmap that facilities contribute to the field of DL for cancer diagnosis. As the field progresses, the upcoming practical DL in cancer will revolve around the incorporation of medicinal imaging and data to uncover biologically significant biomarkers. This mixture can offer unexpected insights, which is exciting. The availability of ironic data for exercise models and medical confirmation of the biological significance of DL-produced visions are important conditions for the widespread deployment of DL in medical settings. When new machineries, such as multiplexed imagery, become widely available, more emphasis will be placed on increasing the quantity and quality of medical statistics labeling and annotation.

## Author Contributions

Conceptualization: AI and AM. Methodology, investigation, and supervision: HM and AM. Analysis: BZ. All authors have read and agreed to the published version of the manuscript.

## Conflict of Interest

The authors declare that the research was conducted in the absence of any commercial or financial relationships that could be construed as a potential conflict of interest.

## Publisher's Note

All claims expressed in this article are solely those of the authors and do not necessarily represent those of their affiliated organizations, or those of the publisher, the editors and the reviewers. Any product that may be evaluated in this article, or claim that may be made by its manufacturer, is not guaranteed or endorsed by the publisher.
